# Anti-inflammatory properties of mutolide isolated from the fungus *Lepidosphaeria* species (PM0651419)

**DOI:** 10.1186/s40064-015-1493-6

**Published:** 2015-11-19

**Authors:** Meet Shah, Sunil Kumar Deshmukh, Shilpa A. Verekar, Akash Gohil, Abhijeet S. Kate, V. Rekha, Asha Kulkarni-Almeida

**Affiliations:** NCE Research Division, Piramal Enterprises Ltd., Mumbai, India; Vellore Institute of Technology, Vellore, India

**Keywords:** Inflammation, Mutolide, *Lepidosphaeria species*, NF-κB

## Abstract

Mutolide an anti-inflammatory compound was isolated from the coprophilous fungus *Lepidosphaeria* sp. (PM0651419). The compound mitigated LPS-induced secretion of pro-inflammatory cytokines TNF-α and IL-6 from THP-1 cells as well as human peripheral blood mononuclear cells (hPBMCs). Mutolide also inhibited secretion of another pro-inflammatory cytokine IL-17 from anti-hCD3/anti-hCD28 stimulated hPBMCs. NF-κB is the major transcription factor involved in the secretion of pro-inflammatory cytokines including IL-17. Mechanistic evaluations revealed that mutolide inhibited induced NF-κB activation and translocation from cytoplasm into the nucleus. However, mutolide did not significantly affect activity of p38 MAPK enzyme, a serine/threonine kinase involved in cell cycle proliferation and cytokine secretion. These results indicate that mutolide may exert its anti-inflammatory effect via NF-κB inhibition. Oral administration of mutolide at 100 mg/kg showed significant inhibition of LPS-induced release of TNF-α from Balb/c mice in an acute model of inflammation. Our results highlight the anti-inflammatory properties of mutolide and suggest that further evaluation in a chronic model of inflammation is required to confirm the potential of mutolide as a druggable candidate for the treatment of inflammatory diseases.

**Electronic supplementary material:**

The online version of this article (doi:10.1186/s40064-015-1493-6) contains supplementary material, which is available to authorized users.

## Background

Inflammation is a complex response to harmful stimuli like microbial infection, endotoxin exposure, damaged cells or irritants. Lipopolysaccharide (LPS), which is produced by Gram negative bacteria, binds to CD14/TLR4/MD2 receptor complex, especially in monocytes, dendritic cells, macrophages and B cells. This results in activation of a complex biochemical cascade that promotes the recruitment of MyD88, activation of protein kinases, recruitment of the adaptor protein TRAF6, and subsequent activation and translocation of NF-κB and AP-1 into the nucleus. NF-κB activation is mediated by phosphorylation of IκB after LPS stimulation culminating in dissociation of the IκB complex leading to translocation of NF-κB into the nucleus wherein it interacts with promoter regions of various genes encoding pro-inflammatory mediators. The activation of NF-κB results in excessive production of pro-inflammatory cytokines such as TNF-α, IL-6, and IL-1β. Several studies have implicated the role of TNF-α, IL-1β, IL-6 in the pathogenesis of a number of inflammatory diseases, such as inflammatory bowel disease (IBD), rheumatoid arthritis, sepsis and mucositis. Since the discovery of TNF-α antibody for the treatment of RA (Feldmann et al. 1996) biologists are targeting TNF-α to reduce inflammatory reactions in the body and restore the cytokine balance. Tociluzumab, a humanized anti-IL-6 receptor antibody has achieved a very good ACR70 response in human clinical trials for RA (Smolen and Maini 2006). Thus, anti-IL-6 treatment is also considered an important strategy for therapeutic intervention. Recent developments with the anti-IL-17 and anti-IL-23 strategies have shown clinical success in the treatment of psoriasis (Griffiths et al. 2010; Leonardi et al. 2012; Papp et al. 2012). The clinical success of these anti-cytokine strategies has increased the focus of pharmaceutical drug discovery on identifying small molecule inhibitors of cytokines such as TNF-α, IL-6, IL-17 and IL-23 (Kulkarni-Almeida et al. 2008).

Historically, the best resources for novel scaffolds have always been natural products. A number of studies have reported that natural products show anti-inflammatory activity by controlling the levels of various inflammatory cytokines or inflammatory mediators including TNF-α, IL-6, IL-1β, NF-κB, JAK, STAT, NO, iNOS, COX-1 and COX-2 (Debnath et al. 2013; Gautam and Jachak 2009). Amongst natural products, fungi are a rich source of chemical diversity (Deshmukh and Verekar 2012; Gunatilaka 2006; Kharwar et al. 2011; Newman and Cragg 2007), and its metabolites are used by the pharmaceutical industry in either the native form or as derivatives (Aly et al. 2011; Bernier et al. 2004). As only a small part of the mycota is known and most fungi produce several unknown metabolites, fungi are still one of the most promising sources for new lead compounds. Fungal metabolites are known potential anti-inflammatory agents and act on targets such as iNOS, NF-κB, AP-1, JAK, STAT, cytokines, cyclooxygenase (COX-1 and COX-2), 3β-HSD, XO and PLA2: Rutilins A and B isolated from *Hypoxylon rutilum*, an inhibitor of NO production(Quang et al. 2006), Gliovirin isolated from *Trichoderma harzianum*, an inhibitor of inducible TNF-α expression (Rether et al. 2007), Panepoxydone isolated from *Lentinus crinitus,* an inhibitor of NF-κB activation (Erkel et al. 1996), Phomol isolated from *Phomopsis* sp. inhibitor of edema in the mouse ear assay (Weber et al. 2004), Ergoflavin isolated from an endophytic fungus of *Mimosops elengi*, an inhibitor of human TNF-α and IL-6 (Deshmukh et al. 2009), are but a few examples.

In our ongoing pharmacological screening program on biodiversity of fungi present in Indian landscape using high throughput screening (HTS); we discovered a remarkable anti-inflammatory activity in extracts/fractions of a fungus Ascomycota, coded as PM0651419. The 14-membered macrolide, mutolide, was isolated by bioactivity guided isolation. This macrolide was first discovered by chemical screening of the culture broth of the fungus F-24′707y, obtained after UV mutagenesis of the wild type strain, which normally produces the spirobisnaphthalene cladospirone bisepoxide (Bode and Zeeck 2000). However, the biological activity of this macrolide was not reported. We describe here the isolation of the active compound mutolide from this culture and demonstrate for the first time the anti-inflammatory properties of mutolide.

## Results

### Phylogenetic tree analysis for sample PM0651419

**Fig. 1:**
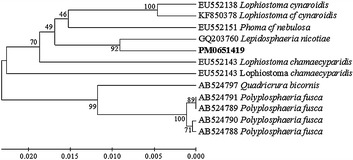
Phylogenetic analysis of the ITS region sequence obtained from sample PM0651419 in comparison with the nearest type strain sequences. The tree was constructed based rRNA gene sequences (ITS region) using the Maximum Composite Likelihood Method

The fungal culture, PM0651419 was identified as *Lepidosphaeria nicotiae* by partial sequencing of the internal transcribed spacer (ITS) region with ITS primers using polymerase chain reaction (PCR). A nucleotide-to-nucleotide BLAST query of the gene bank database (Altschul et al. 1990) recovered GQ203760.1, *Lepidosphaeria nicotiae* as the closest match to the ITS rDNA of PM0651419 (92 %) (Fig. 1). Evolutionary analysis were performed using MEGA 6 (Tamura et al. 2013). The 92 % similarity score does not provide confident species-level identification in the genus *Lepidosphaeria*, hence it was designated simply as a *Lepidosphaeria* sp.

### Isolation and structural elucidation of the compound

**Fig. 2:**
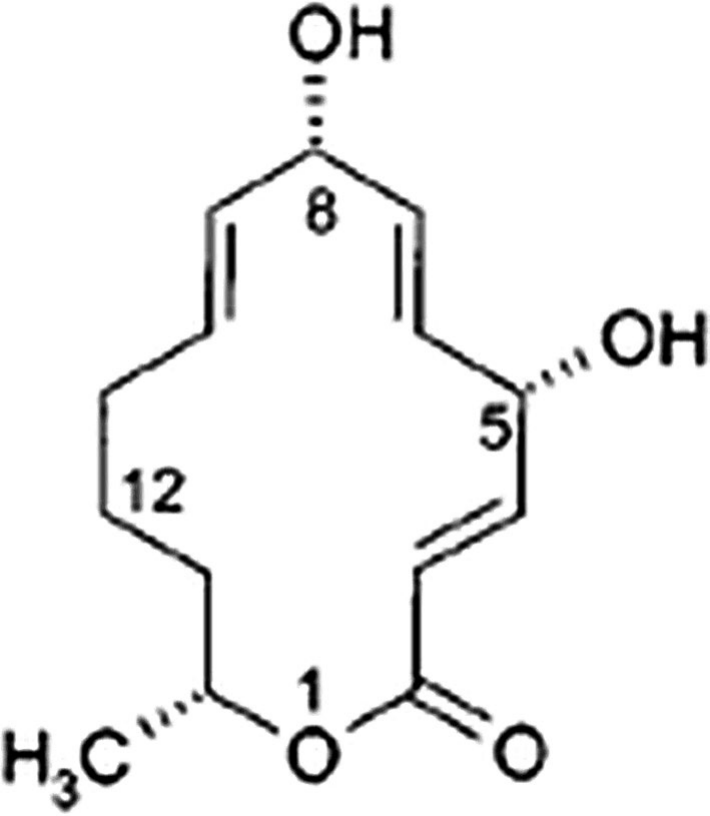
Structure of mutolide

Crude extract of fungus PM0651419 was subjected to vacuum liquid chromatography and organic fraction was generated using 100 % methanol. This organic extract was fractionated by HPLC which included reversed phase column (RP-C18) and water:acetonitrile (0.1 % formic acid) gradient as mobile phase. Fractionation of PM0651419 gave 12 active fractions (1–4: moderate activity, 5–12: potent activity, retention time 0.5–6.5 min.) and only four fractions 5, 7, 9 and 11 were selected for analysis by LCMS, High-Resolution MS. The four fractions/tubes (5, 7, 9 and 11) were mixtures of mutolide and minor unidentified components. All fractions were tested for its effect on induced secretion of TNF-α and IL-6 and active fractions were further purified. Further fractionation on PM0651419-tubes 5 and 7 yielded four active fractions (47 J, 47 K, 47 L and 47 P). High-resolution MS of 47 J, 47 K and 47 P showed mutolide was present as a relatively pure fraction (95 %) (Additional file 1: Figures S1, S2, S3). Anti-inflammatory activity was further confirmed for pure compound. Isolated quantity of pure compound was very low and hence, large scale isolation was carried out for other biological studies. The isolated compound was characterized by spectroscopic data analysis (IR, ^1^H-NMR, ^13^CNMR, and LC–MS). As mentioned in Table 1, all the values were in complete accord with that of the reported values (Bode et al. 2000). Mutolide ((3*E,*5*S,*6*E,*8*S,*9*E,*14*R*)-5,8-Dihydroxy-14-methyloxacyclotetradeca-3,6,9-trien-2-one) (1): soluble in methanol or acetone; insoluble in *n*-hexane or water. M.p. 168 °C—IR (KBr): ν_max_ 3300 cm^−1^ (OH), 1708 (C=O), 1641 (C=C). ^1^H NMR (300 MHz, [D6] acetone) and ^13^C NMR (75.5 MHz, [D6] acetone): (Table 1). Hence the structure of the compound was assigned as mutolide (Fig. 2). Our subsequent data shows the anti-inflammatory potential of mutolide.

**Table 1:** NMR data comparison for mutolide

C-atom	Reported δH (ppm)^a^	Observed δH (ppm)	Reported δc (ppm)^a^	Observed δc (ppm)
2	–	–	168.6	166.244
3	5.81	5.85	118.8	117.383
4	6.7	6.74	152.9	151.744
5	4.89	4.9	70.8	69.425
6	5.41	5.42	131.1	129.744
7	5.77	5.75	135	134.594
8	4.62	4.62	73	71.603
9	5.57	5.66	135.6	134.986
10	5.41	5.56	132.6	130.305
11	1.94	1.97	31.7	30.419
12	1.4	1.4	25	23.656
13	1.52	1.55	35.5	34.138
14	4.98	5	72.8	70.497
15	1.18	1.22	18.9	17.878
5-OH	4.5	4.7	–	–
8-OH	3.96	4.16	–	–

^a^NMR data of mutolide reported in Bode et al. (2000)

### Effect of mutolide on LPS-induced TNF-α and IL-6 secretion from THP-1 cells

**Fig. 3:**
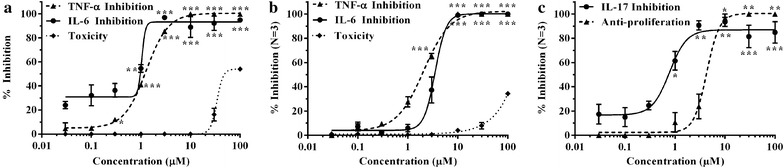
The effect of mutolide on **a** TNF-α and IL-6 secretion LPS-stimulated THP-1 cells after 24 h. Values presented are average of n = 2. Mutolide showed dose-dependent inhibition of TNF-α and IL-6 with IC_50_ being 1.27 ± 0.06 and 1.07 ± 0.02 µM, respectively. CC_50_ for mutolide was 32.16 ± 1.53 µM, **b** TNF-α and IL-6 secretion from LPS-stimulated human peripheral blood mononuclear cells. Values presented are average of N = 3 donors. Mutolide had an IC_50_ of 1.83 ± 0.33, 2.5 ± 0.5 and >100 µM for TNF-α inhibition, IL-6 inhibition and toxicity, respectively, **c** IL-17 secretion from anti-hCD3/anti-hCD28 co-stimulated hPBMCs and its effect on proliferation of anti-hCD3/anti-hCD28 stimulated and unstimulated hPBMCs. Values presented are average of N = 3 donors. Mutolide had an IC_50_ of 0.63 ± 0.04, 4.36 ± 1.02 µM for IL-17 inhibition and proliferation of stimulated hPBMCs, respectively. All data are statistically analyzed by GraphPad Prism version 5.0. *Error bars* represent mean ± SEM. *p < 0.05, **p < 0.01, ***p < 0.001

THP-1 cells are frequently used as a standard system for monocytes due to their similar genetic background. In preliminary experiment, we sought to confirm the anti-inflammatory potential of mutolide. The anti-inflammatory activity was evaluated by the ability to inhibit LPS-induced TNF-α and IL-6 secretion. THP-1 cells were stimulated by LPS after addition of mutolide. After 24 h, supernatant was collected and assayed for TNF-α and IL-6 levels by ELISA. Cytotoxicity was evaluated using MTS. Dexamethasone was used as a positive control. At 1 μM, dexamethasone inhibited the production of IL-6 and TNF-α from THP-1 cells by 90 % and 74 % respectively without significant toxicity on the cells. LPS stimulated THP-1 cells showed 2296 and 84 pg/ml of TNF-α and IL-6 secretion, respectively. As shown in Fig. 3a, mutolide blocked the release of TNF-α and IL-6 by LPS-stimulated THP-1 cells in a dose-dependent manner with an IC_50_ of 1.27 ± 0.06 and 1.07 ± 0.02 µM, respectively, without significantly affecting viability of the cells. IC_50_ for toxicity was 32.16 ± 1.53 µM.

### Effect of mutolide on LPS-induced TNF-α and IL-6 secretion from human peripheral blood mononuclear cells

Further, we sought to evaluate the anti-inflammatory potential of mutolide using primary cells. For this, PBMCs from healthy donors were isolated and stimulated with LPS after adding mutolide. After 5 h of incubation, the supernatant was collected and assayed for measuring TNF-α and IL-6 levels by ELISA. Cytotoxicity of mutolide was assessed by MTS method. The positive control, dexamethasone at 1 µM, inhibited the secretion of IL-6 and TNF-α from hPBMCs by 71 ± 3 and 65 ± 6 %, respectively, without significant toxicity on the cells. Mean TNF-α and IL-6 levels observed from three healthy human donors in this experiment were 4055 and 350 pg/ml, respectively. Mutolide, in this experiment mitigated TNF-α and IL-6 at IC_50_ equivalent to 1.83 ± 0.33, 2.5 ± 0.5 µM, respectively, without affecting viability of hPBMCs up to 100 µM (Fig. 3b).

### Effect of mutolide on anti-hCD3/anti-hCD28 co-stimulated IL-17 release from human peripheral blood mononuclear cells and its effect of proliferation of anti-hCD3/anti-hCD28 co-stimulated human peripheral blood mononuclear cells

To evaluate the effect of mutolide on another pro-inflammatory cytokine IL-17, anti-hCD3/anti-hCD28 co-stimulated hPBMCs were used. PBMCs from healthy donors were isolated and stimulated with anti-hCD3/anti-hCD28 mAb and incubated for 48 h with mutolide. After 48 h incubation, supernatant was collected and assayed for IL-17 levels by homogenous time resolved fluorescence (HTRF) method. The anti-proliferative effect of mutolide on anti-hCD3/anti-hCD28 co-stimulated hPBMCs was measured by the incorporation of ^3^H thymidine in these cells. Mean IL-17 level observed from three healthy human donors was 1906 pg/ml. Mutolide inhibited IL-17 expression with an IC_50_ of 0.63 ± 0.04 µM. Whereas effect on cell proliferation was observed at IC_50_ of 4.36 ± 1.02 µM (Fig. 3c) suggesting that effect on IL-17 was selective and not due to a general cessation of proliferation. Mutolide was further assessed for its effect on activation of the transcription factor RORγt which is centrally involved in IL-17 synthesis (supplementary information). The compound did not affect RORγt activity in a transfected cell line (Additional file 1: Figure S4) suggesting that mutolide may be inhibiting pathways ubiquitously involved in cytokine secretion.

### Effect of mutolide on TNF-α induced NF-κB activation in CEM-κB cells transfected with the κB element

**Fig. 4:**
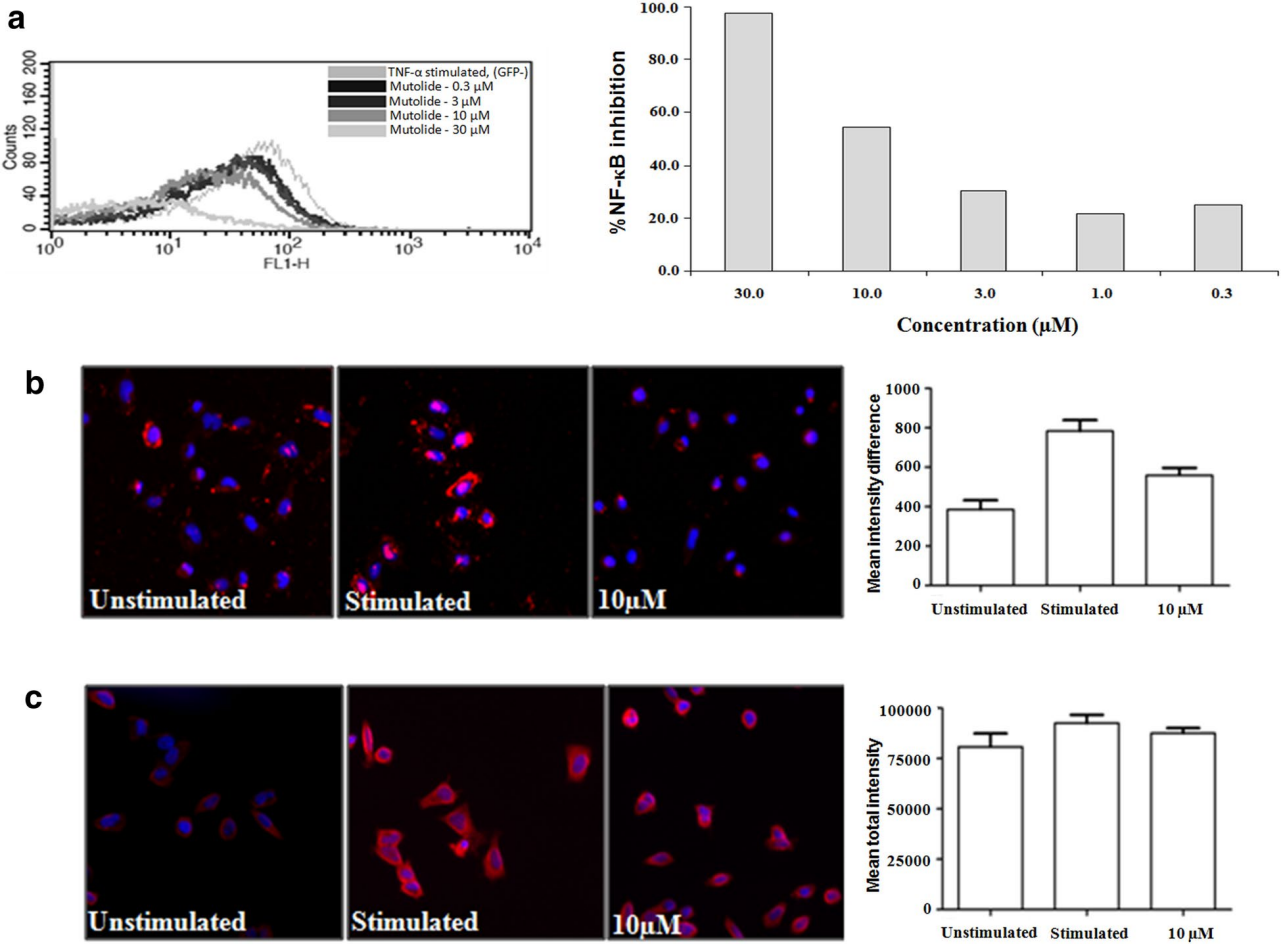
Effect of mutolide on **a** TNF-α induced NF-κB activation in CEM-κB cells transfected with the κB element, **b** translocation of phospho NF-κB and **c** phosphorylation of IκBα. TNF-α induced CEM-κB cells were treated with mutolide and the effect on NF-κB expression at the end of 16 h was determined. Mutolide showed inhibition of TNF-α induce NF-κB activation and the translocation of phospho NF-κB, but not the phosphorylation of IκBα

It is well established that LPS as well as anti-CD3/CD28 signal transduction leads to activation of NF-κB and release of cytokines such as TNF-α, IL-6 by monocytes and IL-17 by T cells. Accordingly to decipher the pathway by which inhibition of induced cytokine secretion in THP-1 cells and hPBMCs was observed; we studied the effect of mutolide on NF-κB transcription using a CEM-κB cell line transfected with the κB binding element. CEM-κB cells were treated with mutolide and subsequently stimulated with TNF-α for a period of 16 h. We observed that mutolide dose dependently prevented activation of NF-κB (Fig. 4a).

### Effect of mutolide on NF-κB activation in HeLa cells

To further elucidate the effect of mutolide on the signaling events involved in NF-κB activation; namely IκB activation followed by p65 translocation, we used TNF-α induced HeLa cells as a tool system. HeLa cells were treated with mutolide at 10 µM followed by stimulation with TNF-α. Mutolide inhibited phospho NF-κB translocation from cytoplasm to nucleus (Fig. 4b) but did not affect phospho IκB activation (Fig. 4c).

### Effect of mutolide on p38 MAPK enzyme

Protein kinases regulate several important functions within cells such as metabolism, cell cycle progression, cell adhesion, etc., p38 MAPK, a serine/threonine kinase, is a critical enzyme in cell proliferation and secretion of cytokines. To evaluate the mechanism by which mutolide inhibits the secretion of LPS-induced TNF-α and IL-6 secretion, it was tested at 5, 25, and 50 µM for its effect on p38 MAPK enzyme activity. SB203580 was used as a positive control. At 1 µM, SB203580 showed 90 % inhibition of p38 MAPK enzyme activity. However, mutolide was not active under similar experiment conditions and did not inhibit p38 MAPK enzyme indicating that the effect of mutolide on cytokine expression and cell proliferation may be independent of the MAPK pathway and may be regulated by action of mutolide on the NF-κB pathway (Table 2).

**Table 2:** Percent inhibition of p38 MAP kinase enzyme activity by mutolide at 5, 25, 50 µM

Compound	Concentration (µM)	% inhibition of p38 MAPK activity
Mutolide	50	26.88
	25	2.25
	5	0.00
SB203580	1	90.03

### Effect of mutolide in vivo production of TNF-α

**Fig. 5:**
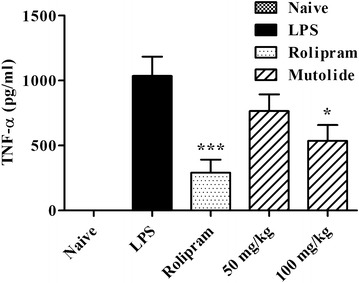
Effect of mutolide on LPS-induced TNF-α production in Balb/c mice. Mutolide was administered orally at 50 and 100 mg/kg followed by LPS stimulation. *Values* presented are average of n = 8 mice. Mutolide significantly inhibited the LPS-induced production of TNF-α from Balb/c mice at 100 mg/kg (p < 0.05 as compared with LPS control). All data are statistically analyzed by GraphPad Prism version 5.0. *p < 0.05, ***p < 0.001

To assess whether mutolide mediated inhibition of pro-inflammatory cytokine observed in vitro could be translated into a meaningful pharmacological effect in vivo, we used an acute model of inflammation. Rolipram, the positive control, significantly inhibited LPS-induced TNF-α production. In this study, oral administration of mutolide at 100 mg/kg significantly inhibited LPS-induced production of TNF-α from Balb/c mice (Fig. 5).

## Discussion

Literature implicates that there are at least 1.5 million fungi in nature. Several fungal metabolites are reported to be inhibitors of iNOS, NFκB, AP-1, JAK, STAT, cytokines, cyclooxygenase (COX-1 and COX-2), 3α-HSD, XO and PLA2 (Deshmukh and Verekar 2012). In natural products based drug discovery, small scale fractionation followed by LC–MS based de-replication has an advantage of screening large number of extract libraries in comparatively a short span of time. The de-replication program along with high throughput screening in different pharmacological indications reveals tremendous valuable information. Here we demonstrate for the first time the anti-inflammatory properties of mutolide isolated from the fungus *Lepidosphaeria* species (PM0651419). There are some differences between mutolide and other macrolides such as azithromycin, clarithomycin, etc. There is concern that that long-term administration of these macrolides can promote bacterial resistance. Non-antimicrobial macrolides (like tacrolimus, pimecrolimus etc.) are in development as potential immunomodulatory therapies. Mutolide has been reported to have a weak antibacterial activity (Pettit 2011). Also, molecular weight of mutolide is significantly lower than other macrolides. Considering this and its anti-inflammatory properties reported in this manuscript, suggests that mutolide if proven efficacious in a chronic model of inflammation, can be used to develop new scaffolds against specific anti-inflammatory targets using modern medicinal chemistry approach.

Exposure of macrophages to bacterial endotoxin or lipopolysaccahride is well known to activate TLR4 mediated signaling cascades that initiate inflammatory gene expression events leading to inflammatory cytokine production. Since, THP-1 cells are widely used cell lines to investigate the function and regulation of monocytes and macrophages (Sharif et al. 2007) and bear resemblance to primary monocytes-macrophages isolated from healthy donors, we utilized these cells for screening and bioactivity guided isolation of mutolide from the fungus PM0651419. Our data clearly indicates that mutolide blocks TNF-α and IL-6 production from LPS-induced THP-1 cells (Fig. 3a). To further verify anti-inflammatory potential using primary cells, mutolide was evaluated for its effect on pro-inflammatory cytokine secretion from LPS-induced human peripheral blood mononuclear cells. Binding of LPS is mediated through LPS binding protein (LBP) and CD14 receptors expressed on the cell surface of cells belonging to the macrophage lineage. LPS induced signal transduction leads to activation of NF-κB and release of cytokines such as TNF-α, IL-6. Mutolide inhibited the secretion of TNF-α and IL-6 from LPS-induced hPBMCs in a dose dependent manner (Fig. 3b).

To evaluate effect of mutolide on other pro-inflammatory cytokines, we looked at IL-17 expression by anti-CD3/CD28 stimulated hPBMCs. Mutolide showed dose dependent inhibition of IL-17 secretion (Fig. 3c) further corroborating its anti-inflammatory potential. RORγt being the transcription factor involved in the differentiation of T cells into IL-17 secreting Th17 cells and currently an engaging industrial target for drug development (Aggarwal and Gurney 2002), we studied the effect of mutolide on RORγt activation. However, mutolide did not mitigate RORγt activation in a reporter assay (Additional file 1: Figure S1) indicating that mutolide may not be exerting its effect on IL-17 secretion via RORγt.

IL-17, a pro-inflammatory cytokine, is found to stimulate the production of many other cytokines such as IL-6, TNF-α, IL-1β, TFG-β, G-CSF, GM-CSF and chemokines such as IL-8, GRO-a, MCP-1 from many cell types. Several studies have shown that IL-17 family is linked to many immune related diseases including RA, asthma, lupus, allograft rejection and psoriasis (Aggarwal and Gurney 2002; Cho et al. 2006; Ju et al. 2008).

Data from THP-1 cells and hPBMCs clearly demonstrated that mutolide significantly inhibited LPS-induced TNF-α and IL-6 secretion. Similarly the compound also abrogated IL-17 secretion from anti-CD3/CD28 stimulated hPBMCs. Cytokines secretion in most cell types is transcriptionally regulated by NF-κB activation. Transcriptional activation of NF-κB target genes in response to extracellular stimuli involves translocation of NF-κB from cytoplasm to nucleus. In the classical pathway, NF-κB protein is bound and inhibited by IκBα. Upon cell stimulation, IKK complex is activated which phosphorylates IκBα. Phosphorylation of IκBα leads to its ubiquitination and proteasomal degradation and thereby NF-κB is released from the IκBα. Active NF-κb is further activated by phosphorylation and translocate to the nucleus. Inside the nucleus, NF-κB either alone or in combination with other transcription factors such as AP-1, ETS and STAT, they induce target gene expression. Aberrant activation of NF-κB is observed in several conditions, most notably, inflammatory tissue injury, where NF-κB controls the gene expression of a variety of pro-inflammatory mediators (Ghosh et al. 1998; Heiss et al. 2001; Tergaonkar 2006).

Many natural products that have been shown to have anti-inflammatory property are known to inhibit NF-κB. Hence, in order to decipher the signaling pathway through which mutolide mitigates cytokine secretion and given the role of NF-κB signaling in cytokine secretion, mutolide was tested for its effect on NF-κB. Our data clearly indicates that mutolide blocks TNF-α induced NF-κB expression in a CEM-κB cell line transfected with the κB element (Fig. 4a). Further, mutolide inhibits TNF-α induced translocation of NF-κB from cytoplasm into the nucleus (Fig. 4b) but has no significant effect on IκB activation (Fig. 4c).

In addition to NF-κB, p38 MAPK enzyme is known to be involved in cell proliferation and cytokine secretion and several p38 MAPK inhibitors are being developed for possible therapeutic effect on autoimmune diseases and inflammatory processes (Goldstein and Gabriel 2005). Here we showed that mutolide does not inhibit p38 MAPK enzyme activity. This indicates that mutolide may exert its inhibitory effect on LPS-induced TNF-α and IL-6 secretion as well as anti-CD3/CD28 induced IL-17 secretion via NF-κB inhibition. Overall our data suggests that mutolide shows promissory anti-inflammatory properties and this activity may be mediated through effects on NF-κB signaling pathway and other transcriptional factors activated by LPS signaling. Further detailed studies are required to elucidate mutolide’s mechanism of action.

Since mutolide is a small molecule which significantly mitigates proinflammatory cytokine secretion and demonstrates inhibition of NF-κB activity, we assessed its potential in abrogating cytokine secretion in vivo. Our data demonstrates that oral administration of mutolide at 50 and 100 mg/kg inhibited LPS-induced production of TNF-α from Balb/c mice (Fig. 5). Since plasma concentrations of mutolide were not evaluated it is not possible to draw a direct comparison to its in vitro effective concentration. However, this in vivo study highlights the potential of mutolide to be effective in mitigating cytokine mediated systemic inflammatory conditions.

Several studies have shown that over expression of cytokines plays a key role in the pathogenesis of autoimmune disease, chronic inflammatory proliferative disease, bone resorption and joint diseases (Feldmann et al. 1996; Papp et al. 2012; Smolen and Maini 2006). The central role of TNF in inflammatory disorders has been demonstrated by the ability of agents that block the action of TNF to treat a range of inflammatory conditions, including rheumatoid arthritis, ankylosing spondylitis, inflammatory bowel disease and psoriasis (Feldmann et al. 1996). Strategies targeting IL-6 and IL-6 signaling lead to effective prevention and treatment of chronic inflammatory diseases (Smolen and Maini 2006). Similarly IL-17 is a crucial cytokine expressed by Th17 cells; triggered by elevated IL-6. Recent data in clinical trials with antibodies directed to IL-17 has shown tremendous success in regression of inflammatory conditions such as psoriasis (Leonardi et al. 2012; Papp et al. 2012). Mutolide is a small molecule which mitigates NF-κB driven inflammatory cytokine secretion by both APCs as well as T cells. NF-κB is the master transcriptional regulator which mediates the secretion of TNF-α and IL-6 in the monocyte–macrophage lineage as well as T cell driven cytokines such as IL-17. Since this transcription factor is ubiquitous, and plays a crucial role in cell differentiation and regulation of specific cellular responses, mitigation of this regulatory factor is considered a good therapeutic option for inflammation (Barnes and Karin 1997). The present studies have clearly indicated that mutolide inhibits pro-inflammatory cytokines by blocking activation of the transcription factor, NF-κB. The molecule when administered orally also abrogates LPS induced cytokine secretion in vivo. Mutolide is a small molecule which exerts systemic anti-inflammatory effects and can therefore be considered as a start-up point for developing new scaffolds against specific anti-inflammatory targets using modern medicinal chemistry approach.

## Conclusions

In this study, we demonstrated the anti-inflammatory potential of mutolide isolated from the coprophilous fungus *Lepidosphaeria* sp. (PM0651419). Our data clearly indicated that mutolide mitigates secretion of pro-inflammatory cytokines TNF-α, IL-6 and IL-17 in different assays. Mechanistic evaluations indicated that mutolide may exert its anti-inflammatory effect via NF-κB inhibition. In an acute model of inflammation, oral administration of mutolide at 100 mg/kg showed significant inhibition of LPS-induced release of TNF-α from Balb/c mice.

## Methods

### Isolation and identification of fungus PM0651419

The culture PM0651419 was isolated from the horse dung samples collected from Rajkot, India, by the method described by Krug et al. using Potato Dextrose Agar (PDA) medium supplemented with 50 mg/L of chloramphenicol (Krug 2004; Krug et al. 2004). The culture was maintained on PDA slant tubes for identification and fermentation purpose.

### Large-scale production of the fungus

A loop full of the well grown culture from slant maintained on Potato dextrose agar (PDA) was transferred to a 500 ml conical flask with 100 ml liquid medium containing soluble starch 1.5 g; soyabean meal 1.5 g; yeast extract 0.2 g; corn steep liquor 0.1 g; glucose 0.5 g; CaCO_3_ 0.2 g; NaCl 0.5 g; glycerol 1.0 g in demineralized water at pH 5.5. This was grown on rotary shaker at 220 rpm for 72 h at 26 ± 1 °C and was used as seed medium. Potato dextrose broth medium (Hi Media) was used for production. The pH of the medium was adjusted to 6.5 prior to sterilization. Twenty-five, 1000 ml flask containing 200 ml of the above medium were inoculated with 1 % of the seed culture and incubated on rotary shaker at 220 rpm for 72 h at 26 ± 1 °C.

### Purification of compound

5 L fermentation broth was filtered through Whatman No. 1 filter paper to remove biomass. The filtrate was passed through the HP20 resin (250 ml bed volume). The organic compound was eluted with 1 L MeOH. The eluate was concentrated on rotary evaporator to remove methanol. The concentrated material was lyophilized to obtain 3.251 g of crude extract. The crude extract was suspended in 100 ml of water and partitioned with ethyl acetate. Evaporation of the ethyl acetate layer yielded a yellow semi solid extract (2.454 mg). Semi solid extract obtain from the ethyl acetate extract was subjected to column chromatography (SiO_2_, 60–120 mesh: CHCl_3_/MeOH gradient 2–20 %). The fractions were tested for its effect on induced secretion of TNF-α and IL-6. The fractions were also monitored by thin layer chromatography (TLC). The pure compound (590 mg) was obtained from fractions eluted with 1.75 % MeOH in CHCl_3._ The purity of compound was determined by HPLC and compound was characterized by spectroscopy.

Normal column chromatography (CC) was performed with distilled commercial-grade solvents. Silica gel (SiO_2_, 200–300 mesh) was used for CC and GF_254_ (30–40 mm) TLCs were procured from Merck. NMR spectra: in acetone d-6recorded on Bruker 300 MHz spectrometer. ESI LC–MS: Bruker Daltonics. Flash chromatography: CombiFlash^®^ Sq 16× Teledyne Technologies Company ISCO attached with UV/VIS detector, RediSep^®^ Flash Column silica 12 g Teledyne ISCO.

### Cell line and THP-1 assay

The human monocytic cell line THP-1 (ATCC) was maintained in RPMI-1640 (GIBCO) supplemented with 2 mM l-glutamine, 100 U of penicillin per ml, 100 mg of streptomycin per ml with 25 mM HEPES and 10 % fetal bovine serum (FBS). Prior to LPS stimulation, 25,000 cells per well were cultured for 24 h in the presence of 10 ng/ml of phorbol 12-myristate 13-acetate (PMA, Sigma-Aldrich). After incubation, non-adherent cells were removed by aspiration, and the adherent cells were washed with RPMI three times. Mutolide or control (0.5 % DMSO) was added to the cells and the plate was incubated for 30 min at 37 °C. Dexamethasone at 1 μM was used as a positive control to assess assay validity. These cells were then stimulated with 1 µg/ml LPS (Sigma-Aldrich) for 24 h. The supernatants were collected and stored at −80 °C until quantification of TNF-α and IL-6 was performed by ELISA using kits from BD biosciences. The cytotoxicity was evaluated by MTS assay (Promega) as per manufacturer’s recommendation.

### Human peripheral blood mononuclear cells assay

Peripheral blood was collected from healthy human donors after informed consent and Independent Ethics Committee approval. Human peripheral blood mononuclear cells (hPBMCs) were isolated using Ficoll-hypaque density centrifugation (1,077 g/ml; Sigma Aldrich) and were suspended in RPMI-1640 media containing 100 U/ml penicillin and 100 µg/ml of streptomycin. For the hPBMCs assay, 1 × 10^6^ cells/ml were plated in a 96-well plate and mutolide was added at eight concentrations ranging from 100 to 0.03 µM. Dexamethasone at 1 μM was used as a positive control to assess assay validity. After 30 min, these cells were then stimulated with 1 µg/ml LPS for 5 h. The supernatants were collected and stored at −80 °C until quantification of TNF-α and IL-6 was performed by ELISA using kits from BD Biosciences. The cytotoxicity was evaluated by MTS assay (Promega) as manufacturer’s recommendation.

For evaluating the effect of mutolide on IL-17 release from hPBMCs, 96-well plates were coated with 1.5 μg/ml anti-human CD3 antibody and 35 ng/ml of anti-human CD28 antibody. For this assay, 1.25 × 10^6^ cells/ml were plated onto these coated plates and mutolide was added at eight concentrations ranging from 100 to 0.03 µM. The supernatants were collected after 48 h and stored at −80 °C until quantification of IL-17 was performed by HTRF assay using kit from Cisbio as per manufacturer’s instruction. In this assay, anti-proliferative effect of mutolide on anti-CD3/anti-CD28 co-stimulated hPBMCs was evaluated by thymidine uptake assay.

### NF-κB transcription assay

The effect of mutolide on NF-κB binding was studied using the CEM-κB cell line. The assay was conducted as per published protocol (Dagia et al. 2010). The CEM-κB cell line was maintained in RPMI containing G418. Cells were stimulated with TNF-α and NF-κB activation was measured as a direct measure of GFP fluorescence. The cells were plated at a density of 50,000 cells/ml and treated with mutolide at various concentrations or 0.5 % DMSO. These cells were then stimulated with or without TNF-α (1 ng/ml; R&D Systems), and the expression of NF-κB was observed after 16 h. The reduction of GFP fluorescence indicates the level of inhibition of NF-κB expression in the cells in the presence of mutolide. BAY 11-7082 was used as a positive control for inhibition of NF-κB activation.

### NF-κB activation in HeLa cells

The specific NF-κB activity was confirmed in HeLa cells stimulated with TNF-α. HeLa cells were seeded at a density of 10,000 cells/well in MEM containing 0.5 % FBS and incubated overnight. Next day, cells were treated with mutolide at 10 µM. After 30 min., cells were stimulated with TNF-α (25 ng/ml) for 5 min followed by fixation with 4 % para-formaldehyde for 15 min at room temperature. Cells were then washed with PBS and permeabilized using 0.5 % Triton X-100 and blocked using BSA. Cells were then stained with anti-phospho NF-κB and anti-IκBα antibodies (Cell Signaling Technology) followed by secondary antibody and DyLight 549 containing Hoechst solution. Cells were then scanned on HCS platform. Data presented is an average of observations recorded per 1000 cells.

### p38 MAPK assay

p38 kinase, ATP, mutolide and ULight-4E-BP1 peptide were diluted in kinase buffer. Mutolide was tested at 5, 25 and 50 µM. SB203580 at 1 μM was used as a positive control. 2.5 µl of each p38 MAPK, mutolide, ULight-4E-BP1 and ATP were mixed in a 384-well plate and incubated at room temperature for 90 min. Kinase reaction was stopped by adding 5 µl of 40 mM EDTA prepared in 1× detection buffer. Subsequently Eu-anti-phospho-eIF4E-binding protein (2 nM) was added and plate was incubated at room temperature. After 60 min, plate was read on Tecan microplate reader in TR-FRET mode (excitation at 620 nm and emission at 665 nm).

### Animals

Male Balb/c mice (8–10 weeks of age, weighing 18–20 g) were housed in individually ventilated cages in a temperature controlled room, with access to water and food ad libitum. Animal experiments were approved by Institutional Animal Ethics Committee of Piramal Enterprises Ltd., NCE Research Division.

### In vivo LPS assay

Mutolide was orally administered to Balb/c mice at 50 and 100 mg/kg in the form of a suspension in carboxymethyl cellulose (CMC; Sigma-Aldrich). One hour later, LPS dissolved in sterile pyrogen-free normal saline was administered i.p. The negative control group received normal saline as an i.p. injection; while all other groups received LPS. Rolipram, a PDE-4 inhibitor, has been shown to significantly reduce serum TNF-α level in the LPS-induced endotoxic shock model and was approved by the Institutional Animal Ethics Committee to be used as an a control in acute model of inflammation. Rolipram was administered at 30 mg/kg. After 2 h, blood was collected and plasma separated by centrifugation at 2000×*g* at room temperature and stored at −80 °C until assayed for mouse TNF-α levels by ELISA.

### Statistical analysis

Statistical analysis was performed using the software package GraphPad Prism. For analyzing differences among multiple (more than two) groups, a single factor ANOVA followed by Dunnett’s multiple comparison tests were used. P values <0.05 were considered statistically significant. All error bars represent standard error of mean.

## Additional files

10.1186/s40064-015-1493-6 HPLC chromatograms of active fractions 47 J, 47 K, 47L, 47 P containing mutolide in pure form and parent PM0651419 (UV 254 nm detection).
10.1186/s40064-015-1493-6 HRMS (Negative mode, ESI-QTOF) of fraction 47 J. [M−H]^−^ = 251.1284 m/z; [M−H+H_2_O]^−^ = 269.1401 m/z. Fractions 47 K and 47 P showed similar HRMS profiles in −ve mode.
10.1186/s40064-015-1493-6 HRMS (Positive mode, ESI-QTOF) of fraction 47 J. [M+H]^+^ = 253.1420 m/z; [M+Na]^+^ = 275.1337 m/z. Fractions 47 K and 47 P showed similar HRMS profiles in +ve mode.
10.1186/s40064-015-1493-6 Effect of mutolide on RORγt activity in a luciferase reporter assay. Mutolide showed inhibition of RORγt reporter activity with IC_50_ of 9.5 µM. CHOK1 cells, stably transfected with RORγt-pFA-CMV plasmid were seeded in a 96-well white plate at a density of 20,000 cells/well in MEM EBS containing 5 % FBS and incubated at 37^0^C overnight. Next day, cells were transiently transfected with pFR luc plasmid in Opti MEM without FBS for 5 h. After 5 h, transfection medium was removed and 200 µl of MEM containing 10 % FBS was added followed by addition of mutolide at 0.03, 0.1, 0.3, 1, 3, 10 and 30 µM. After 18-20 h, cells were lysed and luciferase activity was measured and percent inhibition was calculated.
