# Supplementary figures and images for: Anti-inflammatory properties of mutolide isolated from the fungus Lepidosphaeria species (PM0651419)
Source: Springerplus. 2015 Nov 19;4:706. doi: 10.1186/s40064-015-1493-6 (PMC4653127; doi:10.1186/s40064-015-1493-6)

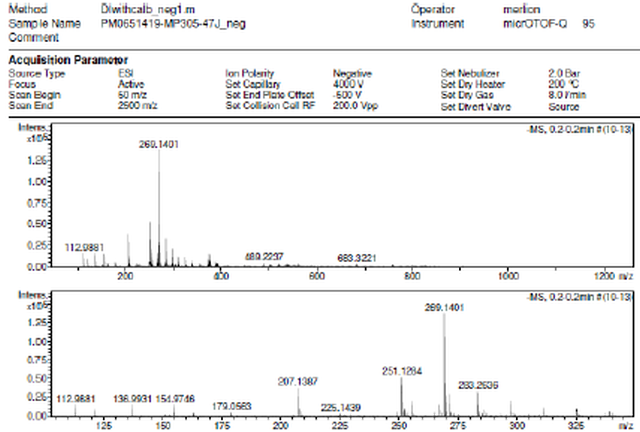

Supplement: Supplementary file 2 — 10.1186/s40064-015-1493-6 HRMS (Negative mode, ESI-QTOF) of fraction 47 J. [M−H]− = 251.1284 m/z; [M−H+H2O]− = 269.1401 m/z. Fractions 47 K and 47 P showed similar HRMS profiles in −ve mode. [file 40064_2015_1493_MOESM2_ESM.tiff]

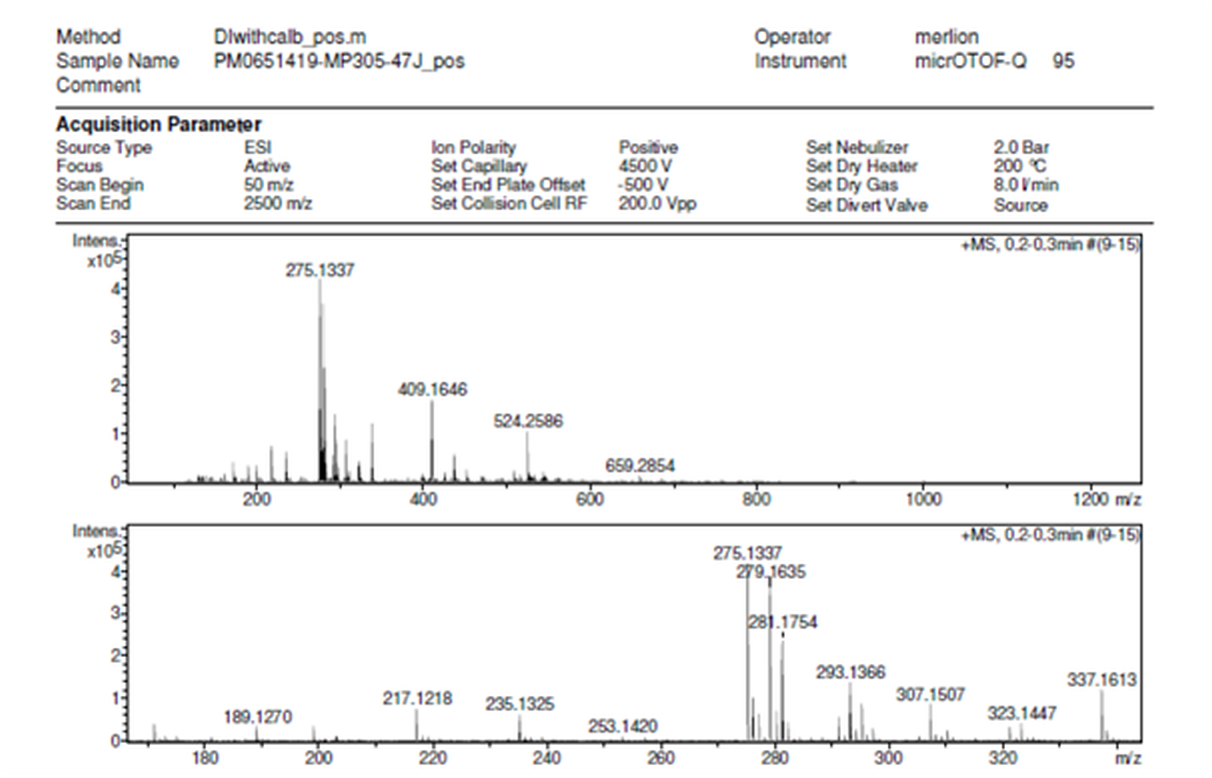

Supplement: Supplementary file 3 — 10.1186/s40064-015-1493-6 HRMS (Positive mode, ESI-QTOF) of fraction 47 J. [M+H]+ = 253.1420 m/z; [M+Na]+ = 275.1337 m/z. Fractions 47 K and 47 P showed similar HRMS profiles in +ve mode. [file 40064_2015_1493_MOESM3_ESM.tiff]

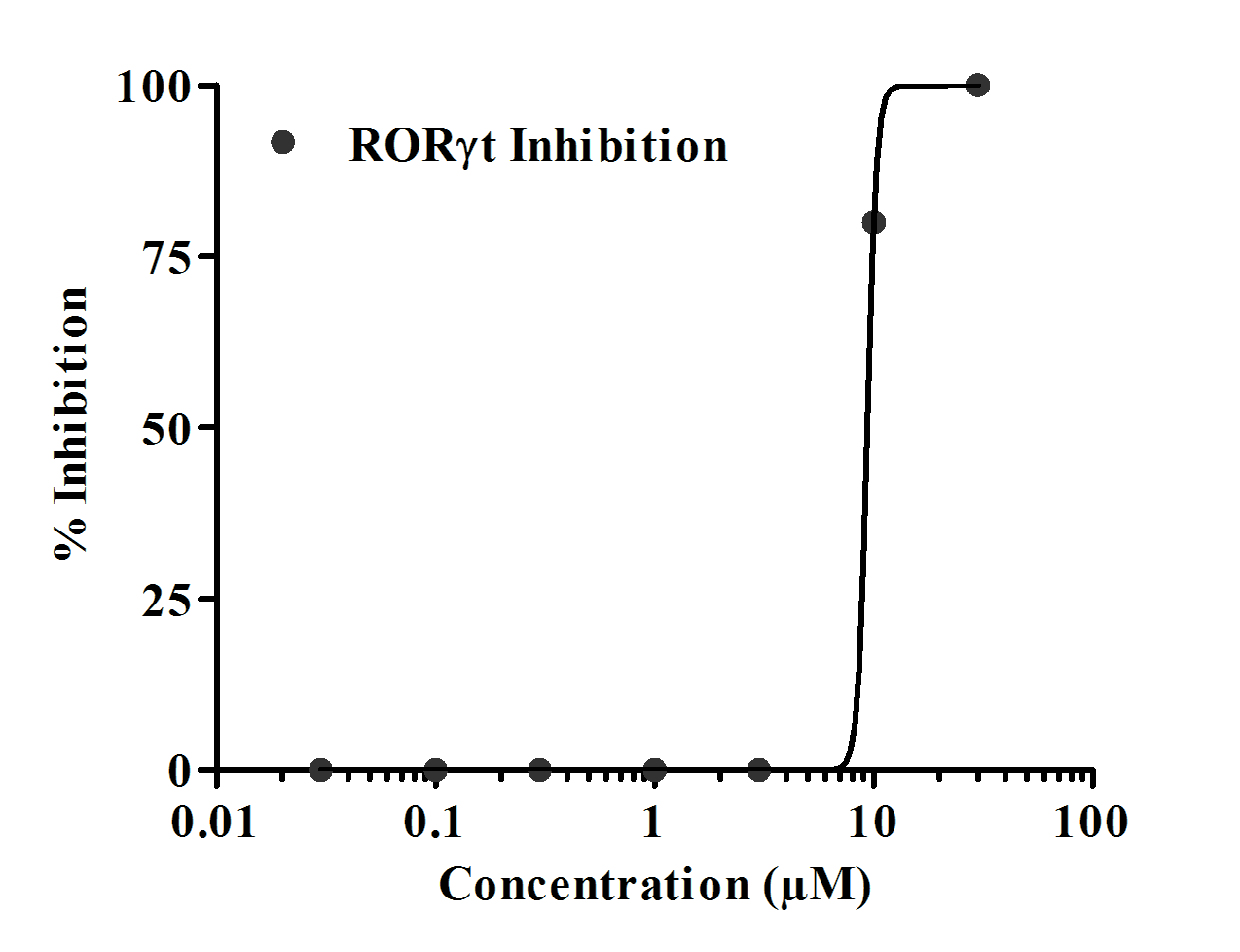

Supplement: Supplementary file 4 — 10.1186/s40064-015-1493-6 Effect of mutolide on RORγt activity in a luciferase reporter assay. Mutolide showed inhibition of RORγt reporter activity with IC50 of 9.5 µM. CHOK1 cells, stably transfected with RORγt-pFA-CMV plasmid were seeded in a 96-well white plate at a density of 20,000 cells/well in MEM EBS containing 5 % FBS and incubated at 370C overnight. Next day, cells were transiently transfected with pFR luc plasmid in Opti MEM without FBS for 5 h. After 5 h, transfection medium was removed and 200 µl of MEM containing 10 % FBS was added followed by addition of mutolide at 0.03, 0.1, 0.3, 1, 3, 10 and 30 µM. After 18-20 h, cells were lysed and luciferase activity was measured and percent inhibition was calculated. [file 40064_2015_1493_MOESM4_ESM.tiff]
